# Enhancement of Photosynthetic Efficiency and Antioxidant Response in Wheat Under Drought Stress by Quercetin–Copper Complex

**DOI:** 10.3390/ijms262110365

**Published:** 2025-10-24

**Authors:** Marta Jańczak-Pieniążek, Dagmara Migut, Tomasz Piechowiak, Maciej Balawejder

**Affiliations:** 1 Department of Crop Production, Faculty of Technology and Life Sciences, University of Rzeszów, Zelwerowicza 4, 35-601 Rzeszów, Poland; 2 Collegium Medicum, Faculty of Biotechnology, University of Rzeszów, Pigonia 1 St., 35-310 Rzeszów, Poland; dmigut@ur.edu.pl; 3 Department of Food Chemistry and Toxicology, Faculty of Technology and Life Sciences, University of Rzeszów, Ćwiklińskiej 1A, 35-601 Rzeszów, Poland; tpiechowiak@ur.edu.pl (T.P.); mbalawejder@ur.edu.pl (M.B.)

**Keywords:** drought, *Triticum aestivum* L., copper-quercetin complex, antioxidant enzymes, relative chlorophyll content, chlorophyll fluorescence, gas exchange

## Abstract

One way to counteract the effects of environmental stresses, including drought, is to use products with growth-promoting properties for plants. Such agents include quercetin, which is known for its antioxidant and photosynthesis-enhancing properties. In the conducted experiment, the influence of the quercetin–copper complex (Q-Cu (II)) treatment, characterized by strong high solubility in water and strong antioxidant properties, was investigated. The pot experiment demonstrated the effect of spraying with Q-Cu (II) solutions (0.01, 0.05 and 0.1%) on wheat plants growing under drought stress conditions. Two treatments of Q-Cu (II) solutions were applied, and chlorophyll content and chlorophyll fluorescence (the maximum quantum yield of photosystem II (PSII) photochemistry (F_v_/F_m_), the efficiency of the water-splitting complex on the donor side of PSII (F_v_/F_o_), and the photosynthetic efficiency index (PI)), as well as gas exchange (photosynthetic network intensity (P_N_), transpiration rate (E), stomatal conductance (g_s_) and intercellular CO_2_ concentration (C_i_)), were measured 1 and 7 days after each treatment. In addition, antioxidant enzyme activity (catalase (CAT), peroxidase (SOD) and guaiacol peroxidase (GPOX)) and reactive oxygen species (ROS) levels were determined. Drought stress caused a decrease in chlorophyll content, and values of parameters F_v_/F_m_, F_v_/F_o_, PI and P_N_, E, g_s_, C_i_, as well as an increase in ROS levels and antioxidant enzyme activity. Exogenous Q-Cu (II) improved photosynthetic indices and modulated redox status in a dose-dependent manner: 0.01–0.05% reduced ROS, whereas 0.1% increased ROS while concomitantly enhancing antioxidant enzyme activities and photosynthetic performance, consistent with ROS-mediated priming. The conducted research indicates the possibility of using Q-Cu (II) as a product to enhance the efficiency of the photosynthetic process under drought stress.

## 1. Introduction

Wheat is of strategic importance worldwide due to its extensive cultivated area of 220.4 million hectares [1], high production and consumption levels, and important nutritional properties. There are possibilities to cultivate wheat under a wide range of climatic conditions and different agricultural production systems [2]. Wheat grain is an important agricultural raw material in terms of energy, carbohydrate, protein, lipid, mineral salts and fiber [3]. Therefore, aiming for its sustainable production on a global scale is of great importance, while taking into account the need to protect the environment and climate [4].

Land plants constantly face environmental stressors throughout their growth and development [5]. These stress factors are generally classified into two major categories: biotic stresses, caused by living organisms such as pathogens, insects, and herbivores, and abiotic stresses, which include drought, salinity, extreme temperatures, heavy metals, and oxidative stress [6]. Among these, abiotic stresses are increasingly recognized as major constraints on crop productivity, especially under the progressing influence of climate change [7,8].

Among abiotic stresses, drought is considered one of the most critical factors limiting agricultural productivity worldwide due to its frequency, intensity, and impact on plant physiology and yield [9]. Water is an essential factor for the sustainable life of all living organisms [10]. The most important consequence of climate change is drought stress, considered a significant and dangerous abiotic stress affecting plants [11,12]. Drought is a physiological form of water deficiency in which the amount of soil water accessible to plants is deficient, and unfavorable effects on plant metabolism [13]. This negatively affects cell proliferation, elongation and specialization, therefore limiting plant growth and development. Drought stress affects the physiological state of plants through a decrease in nutrient supply and cellular toxicity. The effect of drought is loss of turgor, disorganization of enzyme activity and reduced energy transfer from photosynthesis [14,15,16]. Water deficiency, therefore, poses a serious threat to crop production [17] and can cause a quantitative and qualitative decrease in yield by up to 50–70%, affecting crop productivity and reducing the crop’s economic viability [18,19,20,21]. Under drought stress conditions, oxidative stress develops because of elevated levels of reactive oxygen species (ROS) [22]. Normally, ROS produced in plants is in balance with the scavenging mechanism. In the presence of stressful situations, ROS production and removal are disrupted. Increased ROS production results in their accumulation and oxidative damage. Excessive amounts of ROS cause deleterious effects in cells, such as lipid peroxidation in the cell membrane, denaturation of proteins, DNA strand breakage and blocking of photosynthesis [22,23]. Plants in stressful situations in response to biotic and abiotic stresses have evolved a complex set of mechanisms that include morphological and structural changes, drought-resistant gene expression, synthesis of hormones and osmotic regulatory substances, which mitigate the effects of drought stress [13,16,24]. The antioxidant system and osmotic regulation are the major protective systems that ensure plant tolerance to water shortage stress situations. Catalase (CAT), peroxidase (POD), superoxide dismutase (SOD), glutathione reductase (GR), ascorbate peroxidase (APX), and glutathione peroxidase (GPX) are classified as enzymatic antioxidants, while non-enzymatic antioxidants include, for example, vitamins, carotenoids, and phenolic compounds [25,26].

In addition to physiological and biochemical adaptations, drought stress in plants activates complex molecular signaling pathways, including transcription factors (TFs) and stress-responsive genes. These molecular mechanisms interact with hormonal regulation and antioxidant responses, creating an integrated system that increases plant survival and productivity under abiotic stress conditions [27,28,29]. TFs such as Dehydration Responsive Element Binding (DREB), NAC, MYB, WRKY, and bZIP play key roles in regulating gene expression related to osmotic regulation, abscisic acid (ABA) signaling, and ROS detoxification. These TFs initiate the expression of downstream genes that mediate both protective and adaptive responses at the cellular and whole-plant levels [29,30]. Concurrently, a wide range of functional genes, including those encoding late embryogenic abundance (LEA) proteins, aquaporins, and heat shock proteins (HSP), also directly contribute to stress mitigation by stabilizing membranes, maintaining cellular hydration, and protecting protein function under dehydration conditions [31,32]. Although plants have evolved many mechanisms to cope with water stress, their activation often does not prevent the negative impact of drought on yield. Sustainable crop production aims to use safe, environmentally friendly products to help mitigate the effects of drought stress [16]. In recent years, research has been conducted on chemical compounds found in plants that include phytohormones, antioxidants, organic acids and other plant-based secondary metabolites that have phytoprotective and yield-enhancing properties. Phenolic compounds, especially those derived from the shikimate–phenylpropanoid biosynthetic pathway, also enhance plant growth under unfavourable environmental conditions [33]. The application of phytoprotectants such as quercetin is promising compared to traditional farming methods. Quercetin is a natural bioactive substance with antioxidant properties that can potentially modulate physiological processes in plants [34,35]. This compound is a secondary metabolite belonging to a special class of bioactive flavonoids and a subclass of flavonols. Quercetin is found in various plant species (e.g., tea, onion) and plays a key role in stimulating certain physiological processes, such as seed germination, plant growth, and photosynthesis, in both healthy and stressed plants [36,37]. In addition, this flavonoid plays a significant role in balancing levels of ROS and strengthening physiological functions to increase tolerance to environmental stress [35]. However, its use is difficult due to its limited water solubility. Previous studies have confirmed the role of quercetin–metal derivatives in stimulating physiological processes in wheat [38] and maize [39] seedlings growing under optimal conditions and wheat under salt stress [40].

This study aimed to determine the effect of spraying quercetin–copper complex (Q-Cu (II)) solutions on wheat plants exposed to drought stress. The efficiency of the photosynthetic machinery and antioxidant capacity were evaluated. It is hypothesized that the application of quercetin–copper complex, characterized by high antioxidant properties, will improve the tested parameters, which will allow the choice of the optimal concentration for use in wheat cultivation.

## 2. Results

### 2.1. Gas Exchange

**Figure 1:**
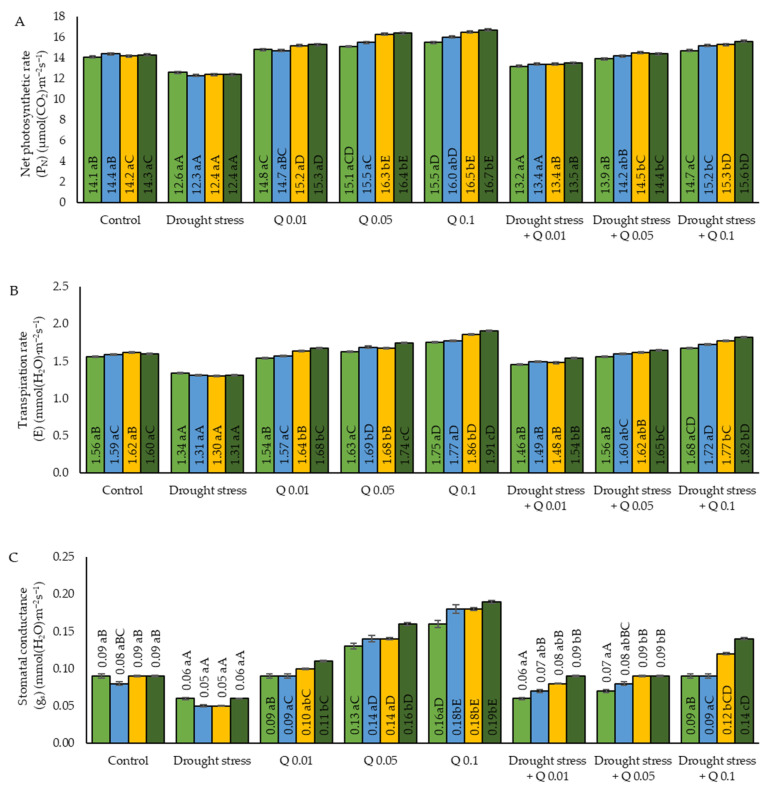

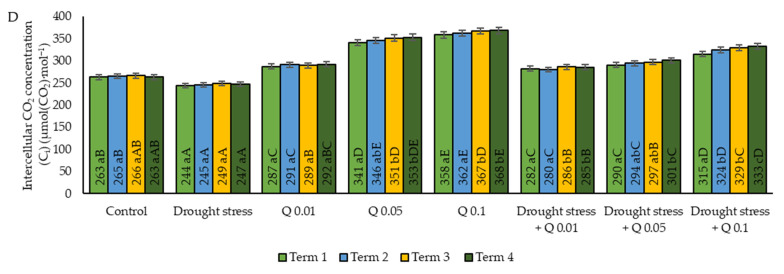
Effect of the concentrations of the Q-Cu (II) solutions on (**A**) net photosynthetic rate (P_N_), (**B**) transpiration rate (E), (**C**) stomatal conductance (g_s_), (**D**) intercellular CO_2_ concentration (C_i_) in wheat leaves grown under draught stress conditions; Term 1—the day after the first spray application, Term 2—seven days after the first spray, Term 3—the day before the second spray, Term 4—seven days after the second spray. Statistical data are expressed as mean ± SD values. Uppercase letters indicate significant differences among averages on consecutive dates of measurement; lowercase letters represent differences among variants on a specific date of measurement (Means ± SD were calculated from *n* = 3 pots; 3 readings were taken in each pot; *n* = 9; *p* ≤ 0.05).

The average P_N_ values of the wheat plants showed significant differences between different experimental variants (Figure 1A). In plants grown under stress conditions associated with water deficiency, significantly lower values of the P_N_ were observed in comparison to the control group. On each measurement date, these values were significantly lower compared to the control group and other variants, confirming the negative effect of water deficiency on photosynthetic activity. The application of the Q-Cu (II) at a concentration of 0.01% resulted in an increase in P_N_ compared to the control group, but the increase was not statistically significant on all measurement dates. In the case of a concentration of 0.05%, the P_N_ values were significantly higher compared to the control group, suggesting greater effectiveness of this concentration. A significant connection was observed between the lower concentration and the control group. The highest P_N_ values were observed in plants in which the 0.01% concentration was applied. Statistical analysis confirmed the significance of the increase in P_N_ compared to the control group and the lower concentrations of the Q-Cu (II). The application of the Q-Cu (II) in the case of plants grown under stress conditions associated with water deficiency yielded varied results. Analyzing the values obtained for the concentration of 0.01%, it was found that the P_N_ values were higher than those in water stress alone; however, the differences between the terms indicate a stabilization of the effect of the Q-Cu (II) during the experiment. In the case of the 0.05% concentration, the mean P_N_ values were significantly higher than those under water stress conditions, and the differences between the terms suggest a stabilization of the effect of the Q-Cu (II) over time. Water stress causes a significant decrease in P_N_ compared to the control group, while application of the Q-Cu (II) leads to a significant increase in P_N_, with the highest values observed in the case of the 0.1% concentration. The highest P_N_ values were found in plants grown under water stress and exposed to a concentration of 0.01%. Statistically, these values differ significantly from those of the control group and the other concentrations of the Q-Cu (II) used.

The applied analyses showed analogous relationships in the context of the data obtained for the E values (Figure 1B). The studies found significant differences depending on the experimental conditions used. The drought stress variant had significantly lower E values compared to the control group. Statistical analysis confirmed that the reduction in transpiration was significant on all measurement dates. The application of the Q-Cu (II) at a concentration of 0.01% did not show significant changes compared to the control group, suggesting a lack of a significant effect of this concentration on transpiration. However, in the case of the application of a concentration of 0.05%, the E values showed an increase compared to the control and these differences were statistically significant. The highest E values were recorded for the concentration of 0.1%, suggesting that this concentration stimulated the transpiration processes the most. The results obtained for plants grown under drought stress and subjected to the application of the Q-Cu (II) were varied. After applying the 0.01% concentration, the E values were higher than under drought stress alone, although still significantly lower compared to the control group, suggesting partial restoration of transpiration activity. The mean E values indicate that the application of the Q-Cu (II) at a concentration of 0.05% resulted in a significant increase in transpiration compared to the variant of drought stress. The highest values were recorded among combinations of drought stress and Q-Cu (II) application for a concentration of 0.1%. Drought stress resulted in a decrease in E values compared to the control, while application of the Q-Cu (II) at different concentrations increased transpiration, especially at higher concentrations (0.1%).

Water stress significantly decreased the g_s_ values, which may indicate a defense mechanism of the plant under water-limited conditions. The application of the Q-Cu (II) increased the g_s_ values, with the highest increase observed at the concentration of 0.1%, both under optimal conditions and during water stress (Figure 1C). Water stress led to a significant decrease in g_s_ compared to the control. The application of the Q-Cu (II) at a concentration of 0.01% did not cause significant changes compared to the control, suggesting that this concentration had no significant effect on the stomatal conductance. A significant increase in g_s_ was observed at a concentration of 0.05%, indicating that the Q-Cu (II) complex at this concentration stimulates the opening of the stomata. The highest g_s_ values were recorded at 0.1% concentration, which confirms the stronger effect of the Q-Cu (II) on the increasing stomatal conductance. Analyzing the results of the interaction of drought stress and application of the Q-Cu (II), a slight increase in g_s_ was found compared to the control variant under drought stress conditions at a concentration of 0.1%, but the values of the parameters analyzed remain significantly lower than in the control sample. The average g_s_ values obtained at a concentration of 0.05% indicate a moderate effect of the Q-Cu (II) on the opening of the stomata under water deficit conditions. The highest increase observed in the combination of drought stress and the application of Q-Cu (II) occurred at a concentration of 0.1%, suggesting that Q-Cu (II) at this concentration may improve the ability of plants to maintain higher stomatal conductance under stress conditions.

The results obtained for wheat plants showed significant differences in the C_i_ value depending on the experimental variant (Figure 1D). In drought stress conditions, the C_i_ value was significantly lower compared to the control group, suggesting limited diffusion of CO_2_ to the leaves, which is a consequence of stomatal closure. The use of the Q-Cu (II), especially at higher concentrations, significantly increased the C_i_ value, which could contribute to an improvement in photosynthetic efficiency. The mentioned complex applied at a concentration of 0.01% led to a moderate increase in the C_i_ value, suggesting partial opening of the stomata and improved availability of CO_2_. In the case of the 0.05% concentration, a significant increase in the C_i_ value was observed, which may indicate an improvement in the intensity of gas exchange. The highest C_i_ values were recorded at the concentration of 0.1%, suggesting that the Q-Cu (II) at this concentration significantly increases the availability of CO_2_ in the leaves. Statistically, these values differ significantly from those of the control group and other applied concentrations of the Q-Cu (II). Drought stress resulted in a significant decrease in C_i_ values compared to the control group, while application of the Q-Cu (II) led to a significant increase in Ci, with the highest values observed at a concentration of 0.1%. In drought stress conditions with the addition of Q-Cu (II), a partial restoration of the C_i_ values to the control level was observed. The results indicate that the application of Q-Cu (II) at a concentration of 0.01% partially alleviated the negative effects associated with drought, but this effect was limited. The moderate increase in C_i_ at a concentration of 0.05% suggests an improvement in CO_2_ availability, but it should be noted that these values were still lower than under optimal conditions. The highest increase in C_i_ was observed in the context of the combination of drought stress and the application of the Q-Cu (II) for the 0.1% concentration, suggesting that higher concentrations of Q-Cu (II) may compensate for the negative effects of the water deficit.

### 2.2. Chlorophyll Content Index (CCI)

**Figure 2:**
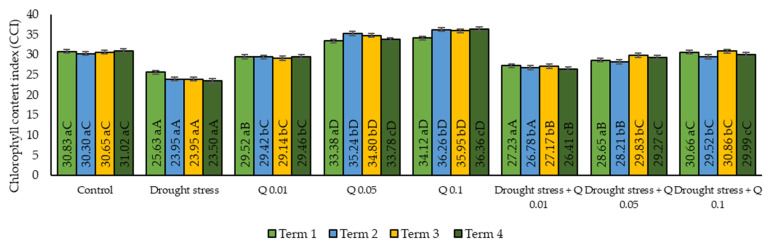
Effect of the concentrations of the Q-Cu (II) solutions on the relative chlorophyll content (CCI) in the leaves of wheat grown under draught stress conditions; Term 1—the day after the first spray application, Term 2—seven days after the first spray, Term 3—the day before the second spray, Term 4—seven days after the second spray. Statistical data are expressed as mean ± SD values. Uppercase letters indicate significant differences among averages on consecutive dates of measurement; lowercase letters represent differences among variants on a specific date of measurement (Means ± SD were calculated from *n* = 3 pots; 10 readings were taken in each pot; *n* = 30; *p* ≤ 0.05).

The mean CCI values showed significant differences between the different experimental variants (Figure 2). Stress from the water deficit led to a significant reduction in the CCI values compared to the control group. Under control conditions, the CCI values ranged from 30.5 to 31.0, while under drought stress conditions, they dropped from 23.5 to 25.9, which was statistically significant. Application of the Q-Cu (II) at a concentration of 0.01% led to a moderate increase in CCI values compared to the control group, suggesting a partial improvement in chloroplast metabolism. In the case of a concentration of 0.05%, a significant increase in CCI values was observed. The highest CCI values were observed at a concentration of 0.1%, suggesting that this level of the Q-Cu (II) had a significant effect on leaf chlorophyll content. The interaction of drought stress and the Q-Cu (II) applied at different concentrations significantly mitigated the negative effects of water shortage in plants. For a concentration of 0.01%, the CCI values indicate that the application of the Q-Cu (II) partially mitigated the negative effects associated with drought, but this effect was limited. A moderate increase in CCI was observed at 0.05% concentration, suggesting an improvement in chlorophyll content, but still lower than under optimal conditions. The highest increase in CCI was observed between combinations of drought stress and the application of the Q-Cu (II) after the application of a concentration of 0.1%.

The application of the Q-Cu (II) led to an increase in CCI values, especially at higher concentrations (0.05% and 0.1%). The highest CCI values were observed at the 0.1% concentration, both under optimal and stress conditions. Under drought stress conditions, the application of the Q-Cu (II) at a concentration of 0.1% significantly mitigated the negative effect of the water deficit on the chlorophyll content.

### 2.3. Chlorophyll Fluorescence

**Figure 3:**
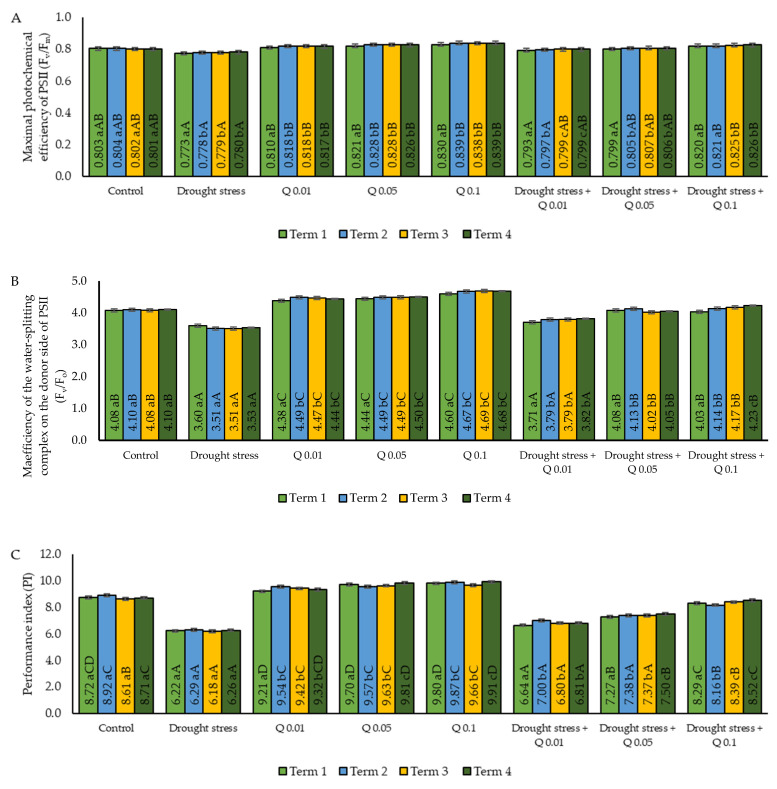
Effect of concentrations of Q-Cu (II) solutions on: (**A**) the photochemical efficiency of PS II (F_v_/F_m_); (**B**) the maximum quantum yield of primary photochemistry (F_v_/F_o_); and (**C**) the performance index of PS II (PI) in leaves of wheat grown under draught stress conditions; Term 1—the day after the first spray application, Term 2—seven days after the first spray, Term 3—the day before the second spray, Term 4—seven days after the second spray. Statistical data are expressed as mean ± SD values. Uppercase letters indicate significant differences among averages on consecutive dates of measurement; lowercase letters represent differences among variants on a specific date of measurement (Means ± SD were calculated from *n* = 3 pots; 10 readings were taken in each pot; *n* = 30; *p* < 0.05).

Drought stress significantly reduced the F_v_/F_m_ index value, indicating a deterioration in the efficiency of the photosynthetic apparatus. Application of the Q-Cu (II) led to an improvement in the value of F_v_/F_m_ in a concentration-dependent manner, both under optimal and stress conditions (Figure 3A). Under control conditions, the F_v_/F_m_ values ranged from 0.801 to 0.804, confirming the correct and undisturbed activity of PS II. Under the influence of drought stress, F_v_/F_m_ values decreased significantly, and these differences showed statistical significance in comparison to the control. At the same time, the lack of significant differences between subsequent measurement dates within the stress variant indicates a permanent and stable reduction in the photochemical efficiency of PS II. The decrease in F_v_/F_m_ values compared to the control by approximately 0.02 to 0.03 suggests the appearance of damage or disorders in the PS II reaction centre. Under optimal conditions, in which there was no water stress, the use of quercetin contributed to the increase in the F_v_/F_m_ values compared to the control, both for concentrations of 0.01%, 0.05% and 0.1%. The F_v_/F_m_ values gradually increased with an increase in the concentration of the Q-Cu (II). The highest values were recorded for the concentration of 0.1%, which is a statistically significant increase and shows the highest efficiency of this concentration. All three concentrations of the Q-Cu (II) differed statistically significantly from the control values, with the effect clearly concentration dependent. Application of the Q-Cu (II) under water stress conditions contributed to partial or full compensation of the negative effect of drought on the F_v_/F_m_ value. Similarly to the application of the Q-Cu (II) alone, the F_v_/F_m_ values gradually increased with increasing concentration of this complex, and the highest values were recorded for the 0.1% concentration. Statistically significant differences between variants suggest that the effectiveness of the Q-Cu (II) in mitigating the effects of stress was clearly dependent on the applied concentration. The values for the 0.1% concentration did not differ significantly from the values under optimal conditions (control), indicating the very high protective efficiency of this concentration. Application of this concentration allowed compensation not only for the negative effect of drought, but also for the exceeding of the control values.

The F_v_/F_o_ parameter is a highly sensitive indicator of the efficiency of primary photochemical processes and the electron transport efficiency in PS II. Data analysis showed that the average values of the F_v_/F_o_ index in four measurement dates for eight experimental variants differed significantly depending on the variant used (Figure 3B). Under control conditions, the F_v_/F_o_ values were 4.08–4.10. Drought stress has resulted in a significant decrease in the value of F_v_/F_o_, which was confirmed by statistical analysis. The decrease in the F_v_/F_o_ value compared to the control indicates a significant reduction in the efficiency of the photosynthetic process. The lack of differences between the measurement dates suggests that the stress effect was stable and maintained regardless of the duration of the experiment. The use of the Q-Cu (II) in the absence of water stress led to a noticeable increase in the F_v_/F_o_ value compared to the control. The application of the Q-Cu (II) at a concentration of 0.01% resulted in a moderate increase in the value of F_v_/F_o_, while a significant increase in this parameter was also observed after the application of a concentration of 0.05%. The highest F_v_/F_o_ values were observed for the 0.1% concentration. All differences were statistically significant compared to those of the control, which proves that the Q-Cu (II) can increase the efficiency of electron transport even under optimal conditions. It should be noted that this effect was concentration dependent: higher concentrations of the Q-Cu (II) resulted in higher F_v_/F_o_ values. In drought-stressed conditions, the application of the Q-Cu (II) complex compensated for the negative effect of water deficiency to varying degrees. For the concentration of 0.01%, an increase in the value of the analyzed parameter was observed compared to stress alone, although it was still lower than under the control conditions. Concentrations of 0.05% and 0.1% differed significantly from the values observed under stress and control conditions, indicating an effective protective effect of the Q-Cu (II), which was also statistically confirmed. After application of the 0.05% concentration, the results of the F_v_/F_o_ values were similar to the control, while for the 0.1% concentration, the values obtained exceeded the control values, suggesting that the highest concentration of the Q-Cu (II) fully compensated for the effects of drought stress and improved PS II functioning. Drought stress significantly reduced the efficiency of primary photochemical processes (F_v_/F_o_), leading to a decrease of more than 0.5 units compared to control conditions. The application of the Q-Cu (II) under optimal conditions significantly increased F_v_/F_o_ values in a concentration-dependent manner. The highest F_v_/F_o_ values were recorded for the 0.1% concentration, both under no stress and under drought conditions, demonstrating the very high effectiveness of this concentration. The Q-Cu (II) at a concentration of 0.1% not only neutralized the effects of stress but also improved the functioning of the photosynthetic apparatus compared to optimal conditions.

The PI reflects the integrated efficiency of PS II, taking into account the efficiency of primary photochemistry, electron transport, and the ability to use them. The values of this index were observed to be different depending on the variants used in the experiment (Figure 3C). Compared to the control sample, stress associated with water deficiency significantly reduced the PI value. Under control conditions, the PI values ranged from 8.61 to 8.92, while under drought stress conditions, they dropped to the range of 6.18 to 6.29, which was statistically significant. Statistical analysis did not show significant differences between terms within the stress variant. The application of the Q-Cu (II) at a concentration of 0.01% resulted in an increase in the PI value in comparison to the control sample. In the case of the 0.05% concentration, an even more pronounced enhancement of the PSII energy efficiency was observed in the presence of the Q-Cu (II). The highest PI values were achieved for the 0.1% concentration. These differences were statistically significant, which confirms the highest effectiveness of this concentration. The application of the Q-Cu (II), especially at concentrations of 0.05% and 0.1%, led to a significant growth in the PI value in comparison to the control sample. Analysing the PI values obtained for the interaction of drought stress and the application of the Q-Cu (II) at different concentrations, it was found that the concentration of 0.01% indicates a moderate improvement concerning stress, although it is still lower than the value in the control sample. In the case of the 0.05% concentration, the PI values increased significantly compared to the stress situation and approached the level of the control samples. The highest values were observed after the application of the 0.1% concentration.

### 2.4. ROS Level

**Figure 4:**
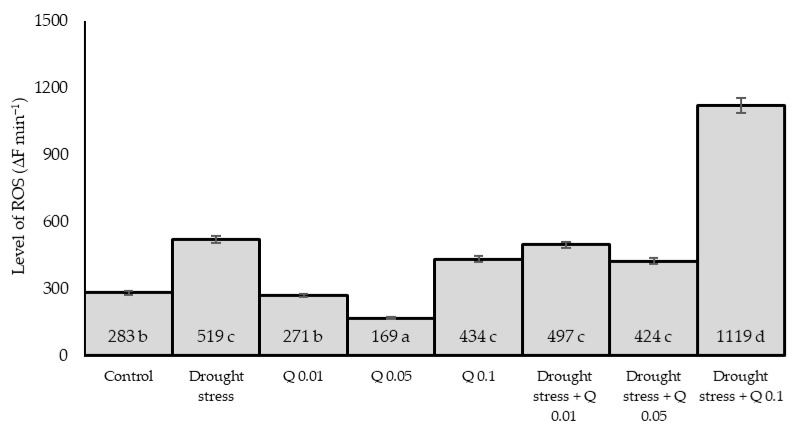
Effect of Q-Cu (II) complex solution concentrations (Q-Cu (II); 0; 0.01; 0.05; 0.1%) and water regime (control conditions—70% WHC; drought—30% WHC) on the level of ROS in wheat leaves (measured at the end point of the experiment). Data are presented as mean ± SD; *n* = 3 pots per combination (biological replicates). Different letters indicate significant differences according to Tukey’s test (*p* = 0.05) between the eight factor combinations (Water × Q-Cu (II)).

**Figure 5:**
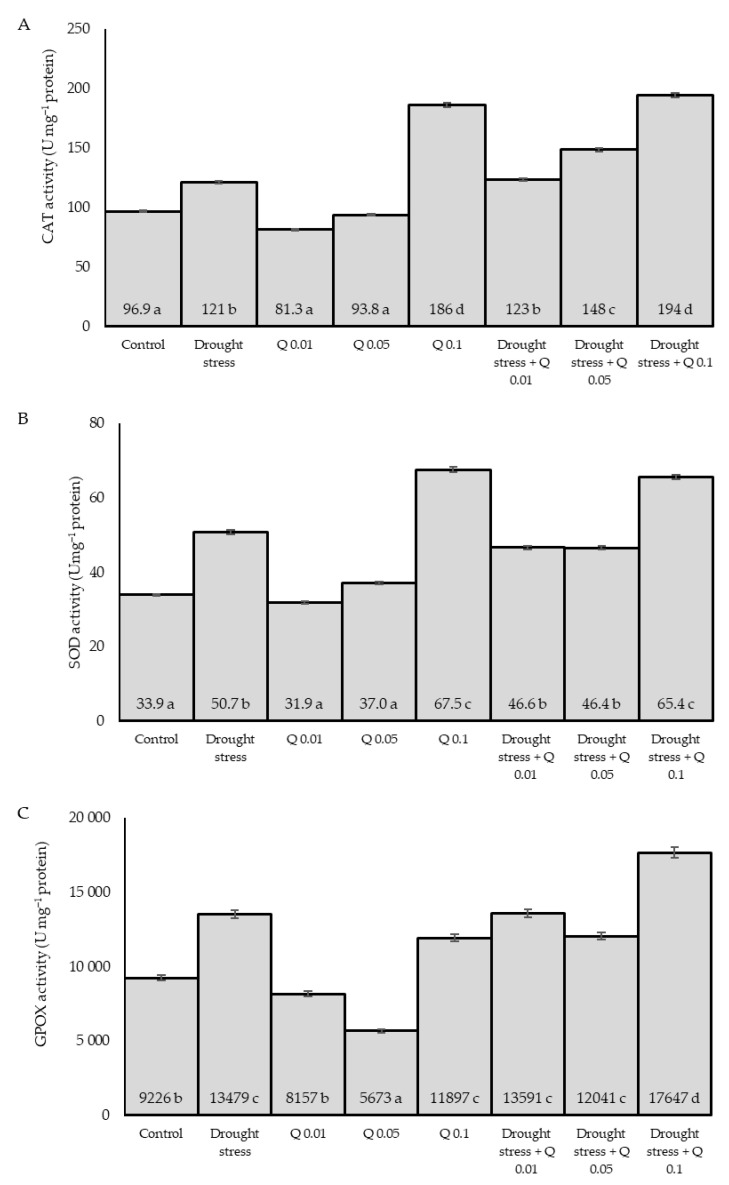
Effect of Q-Cu (II) complex solution concentrations (Q-Cu (II); 0; 0.01; 0.05; 0.1%) and water regime (control conditions—70% WHC; drought—30% WHC) on antioxidant enzyme activities in wheat leaves (measured at the end point of the experiment): (**A**) catalase (CAT), (**B**) superoxide dismutase (SOD), and (**C**) guaiacol peroxidase (GPOX). Data are presented as mean ± SD; *n* = 3 pots per combination (biological replicates). Different letters indicate significant differences according to Tukey’s test (*p* = 0.05) between the eight factor combinations (Water × Q-Cu (II)).

The ROS level varied and depended on the experimental factors used (Figure 4). Drought significantly increased ROS levels compared to the control, reflecting the increased oxidative stress under water stress. The effect of Q-Cu (II) was dose-dependent: 0.01% and 0.05% reduced ROS compared to the corresponding water regime, while 0.1% increased ROS under both control and drought conditions. This pattern indicates a transient, signaling “ROS-burst” at a higher dose of the complex (prooxidant behavior/redox-cycling), which may initiate an antioxidant response and accompanying photosynthetic adjustments (priming/hormesis). Under drought + 0.1%, ROS rose above drought alone, coincident with higher CAT/SOD/GPOX activities (Figure 5), consistent with a hormetic/pro-oxidant signalling response at the highest dose. Application of the derivative at low concentrations reduces the ROS level, with the 0.05% concentration proving to be the most effective. In turn, a high concentration (0.1%) of the Q-Cu (II) derivative increases the ROS level compared to the control group. The combination of drought stress with the Q-Cu (II) derivative at concentrations of 0.01% and 0.05% does not show a significant effect on ROS levels compared to drought stress alone. However, the combination of drought stress with the highest concentration of the derivative (0.1%) leads to a significant increase in ROS levels, suggesting a potentially harmful synergistic effect.

### 2.5. Activity of Enzymes

Drought stress induces an increase in the activity of antioxidant enzymes as a defence response of plants. Compared to the control group, drought stress leads to an increase in CAT activity, suggesting that plants respond to oxidative stress by intensifying the activity of antioxidant enzymes (Figure 5A). The introduction of the Q-Cu derivative (II) shows different effects that depend on the applied concentration, regardless of the presence of drought stress. The lowest concentration (0.01%) caused a decrease in CAT activity, while the concentration of 0.05% slightly increased the value of this parameter, suggesting that in these concentration ranges, Q-Cu (II) has no significant effect on enzyme activity. In turn, the highest concentration of Q-Cu (II) (0.1%) resulted in a significant increase in CAT activity, which may indicate a strong effect inducing the antioxidant response. Under drought stress conditions, Q-Cu (II) supplementation also affected changes in CAT activity. In the case of the combination of drought stress with Q-Cu (II) at a concentration of 0.01%, an increase in CAT activity was observed compared to the variant in which only drought stress occurred. The combination of drought stress with Q-Cu (II) at a concentration of 0.05% further increased CAT activity. The most significant effect was observed when drought stress was combined with the highest concentration of Q-Cu (II) (0.1%), where CAT activity reached the highest level among all groups tested. Drought stress induces an increase in CAT activity as a defence mechanism against oxidative stress. Administration of low concentrations of Q-Cu (II) does not significantly affect enzyme activity, while high concentrations (0.1%) significantly increase it.

Similarly, relationships were observed in the context of SOD activity (Figure 5B). In this case, drought stress leads to a significant increase in SOD activity compared to the control group. The application of the Q-Cu (II) at concentrations of 0.01% and 0.05% does not generate significant changes in the activity of this enzyme compared to the control group. However, the highest concentration of 0.1% results in a significant increase in SOD activity. In drought stress conditions, in combination with different concentrations of Q-Cu (II), various effects were observed. At concentrations of 0.01% and 0.05%, the SOD activity was higher than the control value. The highest concentration of 0.1% in the context of drought stress leads to a significant increase in SOD activity, suggesting an intensified enzymatic response in the presence of stress and high levels of Q-Cu (II).

The study of GPOX activity (Figure 5C) indicates that drought stress results in increased activity of this enzyme. Drought stress significantly increased enzyme activity. The application of the Q-Cu (II) complex at concentrations of 0.01%, 0.05%, and 0.1% shows a varied effect on GPOX activity. The lowest concentration (0.01%) leads to a decrease in GPOX activity, while the 0.05% concentration causes an even greater reduction, suggesting a potential antioxidant effect of Q-Cu (II) at low concentrations. In turn, a 0.1% concentration results in an increase in activity, which may indicate a concentration-dependent effect. Under conditions of drought stress and cooperation of the Q-Cu (II), the activity of GPOX shows clear differences, depending on the applied concentration. In the presence of drought stress, low concentrations of Q do not contribute to reducing the oxidative response of the organism. The highest GPOX activity was observed in the case of a combination of drought stress with a concentration of 0.1%, suggesting that higher concentrations of Q may enhance the defence mechanisms associated with GPOX activity.

## 3. Discussion

Drought resulting from very low rainfall is one of the most common abiotic stresses hampering agricultural activities worldwide. Due to climate change, drought will become a major problem for agriculture, as the frequency of water shortages is predicted to increase [12,41,42]. Drought results in a decrease in water potential and turgor pressure, as well as disturbances in physiological processes [43], including photosynthesis [16,44].

The conducted studies demonstrated the influence of drought stress on the limitation of physiological processes by obtaining unfavorable properties of chlorophyll content and fluorescence parameters, as well as gas exchange. The experiment also determined the level of ROS and the activity of CAT, SOD and GPOX enzymes.

Chloroplasts are organelles essential for photosynthesis. Due to the metabolites synthesized through photosynthesis, and also key proteins involved in metabolic processes, chloroplasts ensure tolerance to different abiotic stresses, including drought. The change and deterioration of chloroplast structure due to drought adversely affects chlorophyll synthesis [43,45]. Chlorophyll is one of the main components of chloroplasts and is continuously metabolized in plants. This most important pigment in photosynthesis can reflect the state of plant growth and the degree of stress. Under drought stress, its content tends to decrease [46]. The conducted experiment demonstrated a significant decrease in chlorophyll content in comparison to the control sample. The obtained results demonstrate that water deficit limits chlorophyll biosynthesis, resulting in a decrease in green pigment content in leaves. The reduction in chlorophyll content may also result from damage to the membrane and structure of chloroplasts, photooxidation of chlorophyll, increased chlorophyllase activity and inhibition of chlorophyll biosynthesis [37,47]. Ashraf et al. [48] and Faisal et al. [45] also showed that the reduction in the concentration of photo-synthetic pigments, including chlorophyll, caused by stress factors can directly limit photosynthetic activity. In addition, the studies of Bhusal et al. [49] demonstrated that the decrease in chlorophyll content in the plant can cause an impairment in the physiological efficiency of plants.

Photosynthesis is a key physiological process involved in the growth and yield of all plants [48,49]. Net photosynthesis directly affects the production of material productivity per leaf area. The rate of photosynthesis and transpiration reduces as the relative water content of the soil decreases [50]. Drought-induced inhibition of photosynthesis affects the decrease in photosynthetic activity, which depends on the type of plant and its developmental stage [44].

The chlorophyll a fluorescence method is a widely used technique to monitor the level of stress caused by damage to the photosynthetic apparatus in various crop species [51,52]. The conducted study showed a decrease in the values of the analyzed chlorophyll fluorescence parameters F_v_/F_m_, F_v_/F_o_ and PI compared to the control. The F_v_/F_m_ index is considered one of the most stable and sensitive parameters for assessing environmental stress. As a result of the drought stress in our study, the values of this indicator were significantly reduced compared to the control. Also, studies by other authors conducted on various plant species confirm the reduction of F_v_/F_m_ values as a result of drought stress [53,54,55]. Shin et al. [56] in a study on lettuce seedlings showed differential effects of drought on chlorophyll fluorescence parameters, the value of which depended on the time of exposure to this stress. The F_v_/F_m_ value, which is an important parameter defining the maximum quantum yield of PSII, showed a significant decrease only at the final stage of the experiment, confirming that the PSII reaction centre was deactivated as a result of photoinhibition when the drought stress reached an extreme stage. However, our study showed no significant differences between measurement dates, indicating a permanent and stable decrease in the photochemical yield of PSII. These differences may be due to species-specific characteristics or the severity of the stress factor [48]. In comparison, a study by Barboričová et al. [57] points to the role of another chlorophyll fluorescence parameter, PI, as more sensitive to a decrease in soil water deficit., The PI index reflects the integrated performance of PS II, considering the efficiency of primary photochemistry, electron transport and the ability to utilize them. The reduction in the value of the PI index shown in our study is accompanied by a significant deterioration in the efficiency of the total photosynthetic system due to water deficit. The F_v_/F_o_ coefficient used to measure the condition and productivity of the electron transport chain in photosynthesis is a method to check how drought stress affects the condition of the plant’s photosynthetic machinery. The F_v_/F_o_ parameter is a highly sensitive indicator of the efficiency of primary photochemical processes and the efficiency of electron transport in PS II. In the control conditions, the F_v_/F_o_ values were 4.08–4.10, which suggests high efficiency of primary photochemical reactions and proper electron transport in PSII. As a result of drought stress, the values of this parameter decreased significantly and ranged from 3.51 to 3.60. The decrease in the F_v_/F_o_ value may indicate an incorrect method of electron transport during photosynthesis and a reduction in the efficiency of this process [58]. The lack of differences between measurement dates suggests that the stress effect was stable and persisted regardless of the experimental duration. The first physiological reaction of plants to drought stress is a decrease in transpiration through stomata closure and a decrease in water loss by the plant. Closure of stomata due to drought results in a decrease in CO_2_ uptake by plants, which affects their photosynthetic activity. Due to the closure of stomata, transpiration also decreases, which limits the adsorption of nutrients from the soil by the roots and their transfer to the higher parts of the plant [58,59,60,61,62]. Water deficiency affects the synthesis of ABA, leading to stomatal closure, thereby contributing to reduced intracellular CO_2_ concentration and impaired photosynthesis [50]. In the studies of Souza et al. [63], Flexas et al. [50], Deeba et al. [64] and Islam [65], the P_N_, E and g_s_ decreased with increasing drought severity. This confirms the strong stomatal response to drought stress also reported by Helm et al. [66]. The closure of the stomata caused by water stress decreases the influx of CO_2_, limiting photosynthesis. As a result, the concentration of CO_2_ in the chloroplast stroma decreases, which causes photorespiration [62]. The decrease in CO_2_ concentration due to stomatal closure reduces the activity of enzymes involved in several dark reactions. A decrease in the light-independent reaction activity may cause a disproportion between light and dark reactions. This leads to the accumulation of ROS in plastids, thus damaging the photosynthetic apparatus [14,67]. Studies by Mathobo et al. [68] and Saeidi et al. [69] showed that drought affected the decrease in stomatal conductance and transpiration, which ultimately reduces plant metabolism and productivity. The activity of the ribulose-1,5-bisphosphate (RuBP) enzyme plays an important role in the photosynthetic assimilation process. There is a relationship between photosynthetic rate and RuBP, which indicates that the decrease in photosynthetic rate is limited by the content of RuBP. Dastborhan and Ghassemi-Golezani [70] also found that drought in wheat reduced stomatal conductance, photosynthetic rate and transpiration rate. Another important enzyme for plant photosynthesis is ribulose-1,5-bisphosphate carboxylase/oxygenase (RuBisCo), which converts CO_2_ into high-energy substances. In the case of drought, the activity of this enzyme is reduced. In this situation, changes in photochemical and biochemical processes were also observed, such as a decrease in the rate of electron transfer and photophosphorylation. The results of this study confirm that stress caused by water deficiency leads to a reduction in the photosynthetic activity of plants by reducing CO_2_ uptake and transpiration, which is manifested in the deterioration of the analyzed gas exchange parameters P_N_, g_s_, E and C_i_. These results suggest that the limitation of water availability leads to a decrease in the intensity of transpiration, probably due to the closure of stomata. This may also indicate a defense mechanism of the plant in drought conditions.

As a result of environmental stresses, plants rapidly accumulate ROS as the first layer of defense [71]. Exposure to stress factors causes overproduction of ROS in plants [72]. In the conducted experiment, a significant increase in ROS level was observed concerning the control. At low concentrations, ROS molecules are not harmful and can act as intracellular signalling agents, triggering plant defense responses. However, in excessive amounts, they can damage macromolecules, including proteins, lipids and nucleic acids, which leads to cell death [73]. In response to excessive ROS accumulation, plants have developed antioxidant enzymatic mechanisms. The conducted research showed a significant increase in the activity of the analyzed CAT, SOD and GPOX enzymes under salt stress conditions compared to the control sample, which proves the plant’s defense reaction. At 0.1% Q-Cu (II), we observed higher ROS levels along with increased CAT, SOD, and GPOX activity and improved photosynthetic parameters. Consistent with the concept of hormesis and possible redox cycling of the Cu-quercetin complex, a higher dose may transiently increase ROS levels, acting as defense signals rather than just damaging molecules. This apparent discrepancy between increased ROS and improved photosynthetic performance can be explained by the dual role of ROS in plants. While excessive accumulation of ROS can damage cellular structures, at low or moderate levels, ROS act as important signaling molecules involved in stress response and regulation of photosynthesis [74,75]. In our study, the observed ~0.1% increase in ROS likely reflects such a signaling role, triggering antioxidant defenses and supporting chloroplast function. This is consistent with recent findings showing that controlled ROS levels can enhance photosynthetic performance by modulating redox signaling and protective mechanisms [76]. SOD is the first defense line in the presence of ROS, which causes the dismutation of O2^−^ radicals to H_2_O_2_. CAT and APX detoxify ROS, help prevent their accumulation in cells and tissues [77,78]. In the case of rice, Harb et al. [79] reported that total SOD activity increased with increasing water deficit. Similarly, increased SOD, CAT, and GPOX activities were shown in barley under drought stress. The antioxidant enzyme SOD plays a role in mitigating drought-induced oxidative stress in crop plants. Previous studies have demonstrated the role of SOD in various crops under drought stress [78,80]. CAT contributes to the decomposition of H_2_O_2_ into oxygen and water, reducing the concentration of this potentially harmful molecule. Previous studies have shown the role of CAT in different crops in response to drought stress. Drought-induced H_2_O_2_ accumulation was associated with reduced soil water content (SWC) in wheat plants. Leaf CAT activity and CO_2_ were only noticeably increased in reaction to the acute drought when SWC fell below 20% [81].

To improve the tolerance of plants to environmental stresses, various approaches are used in practice, including breeding and biotechnological strategies [82]. However, it is necessary to develop simpler and cheaper, environmentally friendly technologies. Because drought stress has become increasingly common, one of the solutions to increase tolerance in crop plants is the use of environmentally friendly products that affect plant metabolism. Such products include phenolic compounds produced by the phenylpropanoid pathway or the shikimic acid pathway as secondary metabolites [35,83,84]. It has been shown that phenolic compounds of plant origin can be externally applied to plants under abiotic stress conditions, which increases their stress tolerance [85]. Phenolic compounds have multiple molecular and biochemical roles in plants, such as antioxidant activity, free radical scavenging, signalling, mediating auxin transport, and plant defense [80]. In plants under abiotic stress, the synthesis and accumulation of phenolic compounds increase [22,35,86]. Phenolic compounds that function as signalling molecules in the regulation of metabolic activity can control water and mineral uptake from roots [87]. Quercetin, a phenolic compound, is a flavonol that has antioxidant and ROS scavenging properties [88]. Previous studies have shown a stimulating effect of this compound on wheat plants [38], maize [39], okra [34] and also under salinity stress conditions in tomato [89] and wheat [40]. Moreover, as a result of the formation of a complex with copper, this compound is characterized by higher antioxidant activity compared to pure quercetin [40,90,91]. In the conducted studies, exogenous application of the Q-Cu (II) showed a positive effect on the analyzed chlorophyll fluorescence parameters, improving the efficiency of PS II in both control and stress conditions. Similar relationships were obtained by Jańczak-Pieniążek et al. [40] in studies on spraying solutions of the Q-Cu (II) on wheat seedlings growing under salt stress conditions. The obtained results proved that concentrations of 0.05% and 0.1% are the most effective in counteracting the effects of salinity. In the conducted studies, the highest values of photosynthesis indices were obtained at a concentration of 0.1%, which suggests its high effectiveness in protecting against water stress. The effect of the complex was concentration dependent, with the highest values achieved at a concentration of 0.1%. These results confirm the effectiveness of the Q-Cu (II) as a factor supporting the functioning of PSII, improving its functioning, and also increasing the efficiency of electron transport, both in optimal and stressful conditions. The effect of the Q-Cu (II) indicates its ability to increase photosynthetic activity, which is achieved by improving stomatal conductance and increasing the availability of CO_2_. The obtained data suggest that the application of the Q-Cu (II), especially at a concentration of 0.1%, can effectively alleviate the negative effects of water deficit on plants. Previously conducted by Arikan et al. [85] research showed the significant role of exogenous application of quercetin on wheat seedlings growing under arsenic stress. In their studies, quercetin increased photosynthetic efficiency and protected photochemical reactions in chloroplasts. This can be explained by reducing oxidative damage by stimulating both enzymatic and non-enzymatic antioxidant activity. Also, studies conducted by Singh et al. [34] showed the effect of quercetin on the antioxidant and photosynthetic apparatus of plants. Increased production of assimilates in plants due to high photosynthetic activity triggers the Krebs cycle. High accumulation of nutrients causes the stomatal opening to enlarge, which increases gas exchange and supports photosynthesis. A significant increase in photosynthesis results in an improved level of assimilates and ultimately contributes to better plant growth, which affects the plant’s achievement of higher yields [34]. In this study, the application of the Q-Cu (II) at the lowest concentration of 0.01% caused a decrease in the activity of CAT, SOD and GPOX enzymes in plants growing in drought conditions. This proves that ROS production is limited due to the action of quercetin. This flavonoid is known for its strong scavenging properties, which result in a decrease in the activity of antioxidant enzymes. Such a connection was also shown in earlier studies by Parvin et al. [89] and Jańczak-Pieniążek et al. [40]. The increase in Q-Cu (II) concentrations, on the other hand, increased the activity of these enzymes, which may indicate a strong effect, inducing an antioxidant response. Strong antioxidant properties of the Q-Cu (II) were also described by Bukhari et al. [90] and Pękal et al. [91]. In drought stress conditions, higher concentrations of Q-Cu (II) strengthened the antioxidant response, which resulted in higher activities of the tested enzymes. The effect of the Q-Cu (II) depends on its concentration. Low concentrations may have a protective effect, reducing enzyme activity, while high concentrations may stimulate enzymatic activity, especially in drought conditions.

The positive effects of Q-Cu (II) observed in this study, particularly in enhancing photosynthetic efficiency and activating antioxidant defenses, can be better understood within the broader context of molecular stress response networks. While enzymatic antioxidants such as SOD, CAT, and GPOX play a direct role in ROS scavenging, their activity is tightly regulated by upstream transcriptional factors. Families such as DREB, WRKY, MYB, and NAC orchestrate the transcription of numerous stress-responsive genes during drought. For example, MbICE1 and MbWRKY50 from *Malus baccata* were reported to modulate ABA signaling and upregulate antioxidant enzyme genes, leading to enhanced drought tolerance [92,93]. Likewise, FvMYB44 in *Fragaria vesca* and VhMYB2 and VhWRKY44 in grapevine contributed to increased ROS scavenging and improved stomatal behavior, suggesting their involvement in adaptation to environmental stress by modulation of both physiological and biochemical pathways [94,95,96,97]. It is therefore likely that the improved photosynthetic and antioxidant performance observed under Q-Cu (II) treatment might be partly associated with the modulation of such transcriptional regulatory pathways. Moreover, functional genes encoding LEA proteins, aquaporins, and heat shock proteins could also be involved, given their role in maintaining cellular homeostasis and protecting metabolic functions during stress. These components, together with Q-Cu (II), may act synergistically to enhance plant resilience under drought conditions [32].

Although the application of Q-Cu (II), particularly at the 0.1% concentration, significantly improved several physiological parameters and enhanced the antioxidant defense system under drought conditions, these findings should be interpreted with caution. The observed biochemical and physiological changes indicate increased stress tolerance; however, they do not necessarily reflect improvements in plant growth or final yield. Enhanced antioxidant activity may represent a protective response to oxidative stress rather than a direct growth-promoting effect. Since this study focused primarily on early physiological responses, morphological traits and yield-related parameters were not assessed. Therefore, further long-term studies under field conditions are required to evaluate whether the positive effects of Q-Cu (II) on plant physiology can be translated into measurable improvements in growth, biomass accumulation, and crop productivity.

## 4. Materials and Methods

### 4.1. Synthesis of the Quercetin–copper (II) Complex (Q-Cu (II))

The Q-Cu (II) was prepared according to the method presented by Bukhari et al. [89], with a modification consisting of increasing the amount of solvent used. 0.001 mol of quercetin was dissolved in 300 mL of methanol. Then, 0.002 mol of solid CuSO4 was added to the system, and the solution was stirred vigorously using a magnetic stirrer for 1.5 h. The formation of the complex was confirmed by analysis of the changes in the UV-VIS spectrum of the reaction mixture. The characteristic yellow-brown solution was filtered, then concentrated and dried using a vacuum evaporator operating at 50 °C and 300 mbar.

### 4.2. Experimental Design

The pot experiment (pot dimensions 11 × 11 × 15 cm, with 3 kg of soil per container) was conducted at the University of Rzeszów (Poland). The experiment was conducted using a commercially available universal horticultural soil mix, characterized by moderate acidity (pH 5.5–6.5), high organic matter content (~30–60%), water holding capacity of approximately 30–70% (*w*/*w*), and low salinity (EC < 1.5 mS/cm) (Table 1). The soil texture is a peat-based mixture with a bulk density ranging from 0.2 to 0.4 g/cm^3^. Typical macronutrient contents include nitrogen (50–150 mg/kg), phosphorus (10–50 mg/kg), and potassium (100–300 mg/kg). These values are representative of the standard soil substrate used in pot experiments. Seeds of winter wheat (*Triticum aestivum* L., cv. ‘Artist’, Deutsche Saatveredelung AG, Lippstadt, Germany) were used in the experiment. ‘Artist’ is a high-yielding winter cultivar with moderate drought tolerance, commonly grown in Central Europe. The experiment was carried out in a growth chamber where the temperature was maintained at 22 ± 2 °C, relative air humidity at 60 ± 3%, photoperiod of 16/8 h (day/night) and maximum light intensity of about 300 µE m^−2^∙s^−1^. In the experiment, the soil in the pots was kept at a moisture level of 70% (control conditions) and 30% (drought stress conditions) of the water-holding capacity (WHC). To apply drought stress in a controlled manner, a gravimetric method was used. Each pot was weighed daily, and water was added to reach a target weight corresponding to the desired soil water content. The target weights were calculated at the beginning of the experiment based on the WHC of the soil substrate. This method ensured uniform application of drought conditions and minimized variability due to uneven water loss. Pot positions in the experiment were chosen randomly every week. The experiment followed a factorial design (2 × 4) in a completely randomized design (CRD) with repeated measures over time. Factor A (water regime): 70% WHC (control) vs. 30% WHC (drought). Factor B (Q-Cu (II) concentration): 0, 0.01, 0.05, 0.1% (*w*/*v*). The experimental unit was the pot (11 × 11 × 15 cm; 3 kg soil). For each water × Q-Cu (II) combination, n = 3 pots (biological replicates) were established. The eight combinations were: (i) Control (70% WHC), (ii) Drought (30% WHC), (iii) Q-Cu (II) 0.01%, (iv) Q-Cu (II) 0.05%, (v) Q-Cu (II) 0.1%, (vi) Drought + Q-Cu (II) 0.01%, (vii) Drought + Q-Cu (II) 0.05%, (viii) Drought + Q-Cu (II) 0.1%. When the plants reached BBCH stage 14 (four unfolded leaves), the spraying procedures were started. The plants were sprayed twice (on the 1st and 10th day) with a solution containing Q-Cu (II) at concentrations of 0.01% (Q1), 0.05% (Q2), and 0.1% (Q3), with a dose of 20 mL for each pot (sprays: days 1 and 10; measurements: Term 1 (day 2), Term 2 (day 9), Term 3 (day 10, before 2nd spray), Term 4 (day 17)). Working solutions of Q-Cu (II) (0.01; 0.05; 0.1% *w*/*v*) were prepared in two steps: Q-Cu (II) was dissolved in a small volume of 96% ethanol (stock solution) and then diluted with deionized water to a final ethanol concentration of ~3% *v*/*v*. The preparation was applied using a handheld sprayer. A uniform spraying methodology was used: An equal amount of solution was introduced into each pot until the point of complete exhaustion of the spray material. A control sample was also filled with deionised water in an equal amount at the same time. The spray was conducted using a manual laboratory sprayer with adjustable flow, where the dose volume was 1.2 mL ± 0.1 during one pressure, and the outlet diameter was 0.6 mm. The physiological parameters of wheat leaves, including gas exchange, relative chlorophyll content, and chlorophyll fluorescence, were monitored at four time points: the day after the first spray application (day two of the experiment—Term 1), seven days after the first spray (day nine of the experiment—Term 2), the day before the second spray (day ten of the experiment—Term 3) and seven days after the second spray (day seventeen of the experiment—Term 4). After taking physiological measurements, the above-ground parts of the plants were harvested, allowing the determination of biochemical parameters, including the level of reactive oxygen species (ROS) and enzyme activity.

### 4.3. Determination of Gas Exchange

The measurement of gas exchange parameters, PN, E, g_s_ and C_i_ was determined using the LC pro-SD photosynthesis measurement system (ADC Bio-scientific Ltd., Herts, UK) equipped according to Jańczak-Pieniążek et al. [40]. The light intensity in the measurement chamber was 1500 mol m^−2^ s^−1^ and the temperature was 28 °C. Measurements were carried out in three replicates per pot.

### 4.4. Determination of Chlorophyll Content Index (CCI)

The CCI was performed in leaves with a CCM 200 metre (Opti-Sciences Inc., Hudson, NH, USA). Measurements were carried out on three fully expanded leaves in ten replicates per pot [40].

### 4.5. Determination of Chlorophyll Fluorescence

The determination of selected chlorophyll fluorescence parameters (the maximum quantum yield of PSII photochemistry (F_v_/F_m_), the efficiency of the water-splitting complex on the donor side of PSII (F_v_/F_o_), and the photosynthetic efficiency index (PI)) was conducted according to the methodology described in the study by Jańczak-Pieniażek et al. [40]. Measurements were taken in 10 replicates per pot.

### 4.6. Measurement of Biochemical Parameters

#### 4.6.1. Determination of ROS Level

The fluorometric method using 2′,7′-dichlorodihydrofluorescein diacetate (H_2_DCF-DA) was employed to determine the level of reactive oxygen species (ROS) in the extracts [98].

Briefly, frozen tissue (1 g) was homogenised in 4 mL of ice-cold 50 mM phosphate buffer (pH 7.4), and the homogenate was centrifuged at 10,000× *g* for 30 min at 4 °C. Then, 100 µL of the resulting supernatant was blended with 1000 µL of phosphate buffer and 50 µL of 2 mM H_2_DCF-DA. After a 30 min incubation at 37 °C, fluorescence was measured at 529 nm (excitation at 504 nm). The results were expressed as the increase in fluorescence per gram of tissue per minute.

#### 4.6.2. Determination of Antioxidant Enzyme Activity

Frozen plant tissue (1 g) was homogenised in 4 mL of chilled 0.9% NaCl solution containing 2% polyvinylpyrrolidone, 0.05% Triton X-100, and a protease inhibitor cocktail to identify the activity of SOD, CAT and GPOX. The next process was to centrifuge the homogenates at 10,000× *g* for 30 min at 4 °C. The resulting supernatant was collected for further analysis. The SOD activity was measured using the adrenaline method described by Piechowiak and Balawejder [98], with minor modifications. Briefly, 10 µL of extract was mixed with 95 µL of carbonate buffer (pH 10.2) and 5 µL of 10 mM adrenaline. The increase in absorbance was measured over 5 min at 490 nm. One unit of SOD activity was defined as the amount of enzyme required to inhibit adrenaline oxidation by 50% [99]. The CAT activity was determined using a method based on the measurement of residual H_2_O_2_ after an enzymatic reaction with ammonium metavanadate [99,100]. For this purpose, 10 µL of extract was added to the wells of a microplate, followed by 40 µL of 50 mM phosphate buffer (pH 7.0) and 100 µL of 10 mM H_2_O_2_ (prepared in phosphate buffer). After 5 min of incubation at 37 °C, the reaction was stopped by adding 100 µL of 10 mM ammonium metavanadate (in 0.5 M H_2_SO_4_). Following a 10 min incubation, the absorbance of the solution was measured at 452 nm. One unit of CAT activity was defined as the amount of enzyme that decreases the absorbance of the reaction mixture by 0.01 units within 1 min of incubation. To determine the GPOX activity, 5 µL of enzyme extract was mixed with 100 µL of a reaction mixture containing 50 mM phosphate buffer (pH 7.0), 19.95 mM H_2_O_2_, and 9.45 mM guaiacol. The kinetics of absorbance changes were measured immediately after the addition of the reaction buffer for 5 min at 470 nm [100]. One unit of GPOX activity was defined as the amount of enzyme that increases the absorbance by 0.01 units within 1 min. Enzyme activity was normalized to mg of protein, the amount of which was determined by the Bradford method [101].

### 4.7. Statistical Analysis

Data were analysed in TIBCO Statistica 13.3.0. For P_N_, E, g_s_, C_i_, CCI, F_v_/F_m_, F_v_/F_0_ and PI we used a two way repeated measures ANOVA: fixed factors Water (2 levels) and Q Cu (II) (4 levels) with Time (4 levels: Term 1–4) as the within subject factor, Pot as the subject (random effect). We tested main effects and interactions (Water × Q, Water × Time, Q × Time, Water × Q × Time). Sphericity was checked (Mauchly); when violated, Greenhouse–Geisser correction was applied. Normality of residuals (Shapiro–Wilk) and homogeneity of variance (Levene) were verified; data were transformed when needed. For ROS, CAT, SOD and GPOX (single time point), we used two way ANOVA (Water, Q Cu (II), Water × Q). Post hoc comparisons were performed with Tukey HSD on estimated marginal means at α = 0.05.

## 5. Conclusions

**Figure 6:**
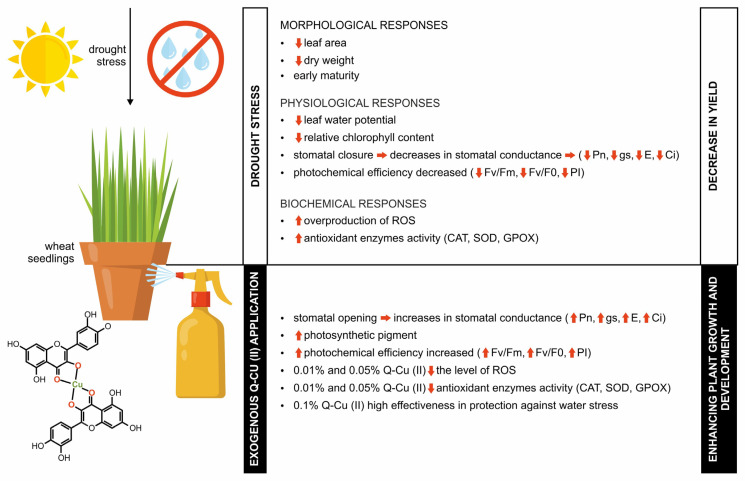
A schematic illustration of the stimulatory action of Q-Cu (II) application in improving morphological, physiological and biochemical responses under drought conditions. Upward arrows (↑) indicate an increase in the parameter value or activity, whereas downward arrows (↓) indicate a decrease.

This study has shown that drought stress has resulted in the deterioration of physiological parameters, indicating a decrease in the efficiency of the photosynthesis process. As a result of this stress, an increase in the level of ROS and the activity of CAT, SOD and GPOX enzymes was also noted. Exogenous application of Q-Cu (II) solutions enhanced the parameters’ values in plants growing in optimal conditions as well as those subjected to drought stress (Figure 6). The highest values of the studied indicators were obtained at a concentration of 0.1%, which suggests high effectiveness in protection against water stress. In drought conditions, the Q-Cu (II) not only compensated for the negative effects of drought but also improved the functioning of the photosynthetic apparatus, exceeding the control values at higher concentrations.

The conducted studies indicate the possibility of using the Q-Cu (II) as a product to support the efficiency of the photosynthesis process under stress conditions. The use of such products in the cultivation of plants increases their productivity, especially in environments exposed to water deficit, which will reduce the threat to global food security. The results of the studies will be useful in particular for farmers interested in developing environmentally friendly products of increasing plant tolerance to environmental stresses, including drought stress. Although the application of Q-Cu (II) improved physiological responses and antioxidant activity under drought stress, further research is needed to determine whether these changes result in improved growth or yield. Future studies should include morphological and agronomic traits to fully assess the practical benefits of this treatment.

## Figures and Tables

**Table 1:** Soil Characteristics of Standard Universal Horticultural Soil Mix.

Parameter	Typical Value	Unit
Soil Texture	Peat-based mix	–
**pH**	5.5–6.5	–
**Water Holding Capacity**	30–70	% (*w*/*w*)
**Field Capacity**	~30–40	% (*w*/*w*)
**Permanent Wilting Point**	~15–20	% (*w*/*w*)
**Bulk Density**	0.2–0.4	g/cm^3^
**Organic Matter Content**	30–60	%
**Electrical Conductivity (EC)**	<1.5	mS/cm
**Macronutrients (N, P, K)**	N: 50–150, P: 10–50, K: 100–300	mg/kg

## Data Availability

The data presented in this study are available from the corresponding author upon reasonable request.

## Abbreviations

The following abbreviations are used in this manuscript:

Q-Cu (II)	quercetin–copper complex
ROS	Reactive Oxygen Species
PSII	Photosystem II
F_v_/F_m_	The maximum quantum yield of PSII) photochemistry
F_v_/F_o_	the efficiency of the water-splitting complex on the donor side of PSI
PI	Photosynthetic efficiency index
P_N_	photosynthetic network intensity
g_s_	stomatal conductance
E	transpiration rate
C_i_	intercellular CO_2_ concentration
CAT	catalase
SOD	peroxidase
GPOX	guaiacol peroxidase
ABA	abscisic acid
TFs	transcription factors
DREB	Dehydration Responsive Element Binding
LEA	late embryogenic abundance
HSP	heat shock proteins
